# Early and short-segment anterior spinal fusion for cervical spinal cord injury without fracture and dislocation can achieve more significant neurological recovery: a retrospective study based on the current medical system in southern China

**DOI:** 10.1186/s13018-019-1487-0

**Published:** 2019-12-05

**Authors:** Xiaoping Mu, Zhuhai Li, Yufu Ou, Jianxun Wei

**Affiliations:** Department of Orthopaedics, The People’s Hospital of Guangxi Zhuang Autonomous Region, Nanning, 530021 China

**Keywords:** Cervical spinal cord injury without fracture and dislocation, CSCIWFD, SCI, Early surgery, Timing of surgery, Fused segments

## Abstract

**Objective:**

The purpose of this study was to investigate the effects of the number of fused segments, the timing of surgery and their interaction on the prognosis of patients with cervical spinal cord injury without fracture and dislocation (CSCIWFD), and to determine the appropriate time restrictions for early surgery in CSCIWFD patients based on the current diagnosis and treatment system in southern China.

**Methods:**

CSCIWFD patients who underwent anterior cervical decompression and internal fusion (ACDF) from January 2012 to June 2017 were selected. The patients were grouped according to the timing of surgery and the number of fused segments and evaluated based on their American Spinal Injury Association (ASIA) score, ASIA impairment scale, and Japanese Orthopaedic Association (JOA) score before and after surgery. SPSS22.0 software was used for the statistical analysis.

**Results:**

The ASIA score, JOA score, and ASIA impairment scale in all follow-ups were significantly higher than before surgery (*p* < 0.05). The ASIA and JOA scores at 6, 12, and 24 months after surgery of the patients who underwent ACDF within 72 h were significantly better than those of the patients who underwent ACDF after 72 h (*p* < 0.05). There were significant differences in postoperative ASIA and JOA scores at 12 and 24 months between the short-segment and three-segment fusion groups (*p* < 0.05). The results of the interaction between the surgical timing and the number of the fused segments showed that the postoperative ASIA and JOA scores at 6, 12, and 24 months were significantly higher in the patients who underwent early short-segment fusion than in those who underwent delayed short-segment fusion (*p* < 0.05). However, no statistically significant difference was found between early and delayed surgery in the patients who underwent three-segment fusion (*p* > 0.05).

**Conclusion:**

ACDF is safe and effective for the treatment of CSCIWFD. For patients with single- or double-segment injury, early (within 72 h) ACDF is associated with a more satisfactory prognosis. Due to the limitation of the small sample size, we cautiously recommend that 72 h can be used as a time limit for early surgery for CSCIWFD patients in regions where earlier surgery cannot be provided by the current diagnosis and treatment system.

## Introduction

In recent years, with the development of transportation and aerial work, increasing numbers of patients have suffered from traumatic cervical spinal cord injury (SCI). As one form of traumatic cervical SCI, the incidence of cervical spinal cord injury without fracture and dislocation (CSCIWFD) in adults is very high due to preexisting cervical degeneration and that the actual rate is underestimated [1, 2]. Trauma is a major cause of CSCIWFD. The manifestations of SCI may vary due to the involvement of different injury mechanisms and trauma types. Due to a lack of significant positive findings on radiography and other imaging examinations, CSCIWFD is easily misdiagnosed as post-traumatic spinal shock or concussion; consequently, the best time for treatment may be missed [3].

For these patients, conservative treatment is the main option, but problems such as a long treatment cycle, a high complication rate, high mortality, and long-term functional decline are common [4, 5]. Therefore, most clinicians prefer early surgical intervention [2]. Current studies indicate that changes in cervical spinal cord signals on cervical magnetic resonance imaging (MRI) strongly indicate that surgical treatment is required [6]. For patients with anterior spinal cord compression limited within 3 segments, the anterior approach is preferred [7].

For patients with CSCIWFD, anterior cervical decompression and fusion (ACDF) is mainly performed to explore the ruptured disc, remove the compressed tissue anterior to the spinal cord, reconstruct the cervical spine, and provide conditions for spinal cord functional recovery and cervical segment stabilization. However, no consensus has been reached regarding the effects of the number of fused segments on postoperative efficacy and adjacent segment degeneration (ASD) [8] or the optimal surgical timing [2, 9, 10] in the academic community.

Previous studies mainly focused on the impact of a single factor, the timing of surgery, on the prognosis of patients with CSCIWFD. However, the factors affecting the prognosis of these patients are often complex. We think that the number of fused segments and the timing of surgery may have an interactive effect on the prognosis of CSCIWFD patients. In addition, the current medical system in our region cannot guarantee early surgery according to the current definition of early surgical timing (e.g., 8 h or 24 h). Therefore, we carried out this research to (i) explore the effects of the number of fused segments and the timing of surgery on the prognosis of CSCIWFD patients, that is, to answer the clinical question of the best timing for these patients to undergo ACDF for different numbers of segments, and (ii) determine the appropriate early surgical time limit for CSCIWFD patients according to the current diagnosis and treatment system in southern China.

## Material and methods

### Patient selection

The data of patients with CSCIWFD who underwent ACDF from January 2012 to June 2017 were reviewed. The inclusion criteria were as follows: (i) patients with a clear history of trauma, such as a fall from height or a car accident; (ii) patients with symptoms and signs consistent with the vertebral level of injury, such as sensory and/or motor dysfunction; (iii) cervical images revealing varying degrees of cervical degeneration and abnormal signal of the lesion site, but no obvious fracture or dislocation; (iv) the injured segment located in the C3–C7 region and a cervical spinal canal index greater than 0.75; (v) met the ACDF operative indicators: the segment with T2 hyperintensity in the spinal cord, the segment with T2 linear hyperintensity in the disc, or the segment with spinal cord compression; (vi) patients with complete data at each follow-up point (preoperative; postoperative 1, 6, 12, and 24 months; and final follow-up).

The exclusion criteria were as follows: (i) patients with an uncertain diagnosis or meeting only one of the clinical and imaging criteria; (ii) patients with posterior spinal cord compression caused by ossification of the posterior longitudinal ligament or the ligamentum flavum; (iii) patients who could not tolerate surgery due to aging or other concurrent diseases; (iv) patients with severe osteoporosis and a risk of postoperative collapse or failed internal fixation; (v) patients who were unable to cooperate due to mental illness or other reasons; and (vi) patients with a previous history of cervical spine surgery.

The patients agreed with the treatments given by the doctor, with a signed informed consent form provided before surgery. This study complied with the principles of the Declaration of Helsinki and was approved by the Ethics Committee of People’s Hospital of Guangxi Zhuang Autonomous Region.

### Perioperative management

Before surgery, organ function was assessed, and related cervical imaging examinations were completed. Information regarding the procedure and recovery was discussed with the patients prior to surgery. The target segments were identified according to clinical manifestations and imaging examination results. The detailed criteria are (1) MRI suggesting disc herniation at the level corresponding to the spinal cord injury or (2) MRI suggesting no disc herniation at the level corresponding to the spinal cord injury but a T2-weighted image showing hyperdense signals in the intervertebral disc or anterior annulus fibrosus of the disc. The target segments for surgery were determined based on evidence of either (1) or (2) in addition to corresponding symptoms and signs. Skin preparation in the operative field was performed, and prophylactic antibiotics (30 min before surgery) were administered.

After successful anesthetic induction with tracheal intubation, the patient was placed in the supine position with the cervical spine tilting backward. A horizontal incision was made on the ride side at the level of the thyroid cartilage through the tissue layers to access the anterior vertebrae. The intervertebral disc was identified after dissection of the longus collis muscle, and the injured intervertebral space was located by fluoroscopy. After retractor needles and a distractor were placed, a nerve stripper was routinely used to explore the integrity of the intervertebral disc. A scalpel was used to incise the annulus fibrosus, and the nucleus pulposus was removed. A scraper was used to curette the cartilage endplate, the residual nucleus pulposus, and the annulus fibrosus until reaching the posterior longitudinal ligament. After cage testing, an interbody fusion cage and autologous bone powder were placed into the intervertebral space. The procedure for multisegment fusion was the same as described above. A steel plate was placed in the proper position and locked after C-arm fluoroscopy showed that the plate was in a good position. A drainage tube was placed, and the wound was closed in layers.

Postoperative routine treatments, including neuro-nutrition agents, diuretics, and analgesics, were applied, and prophylactic antibiotics were continued until 48 h after surgery. The drainage tube was removed when the drainage volume was less than 50 ml. Follow-up fluoroscopy of the cervical spine was performed. One day after surgery, an experienced technician helped with rehabilitation exercises at the bedside.

### Observation indicators

i) American Spinal Injury Association (ASIA) score and ASIA impairment scale;
ii) Japanese Orthopaedic Association (JOA) score of the cervical spine: The JOA score was determined according to the patient’s movement, sensation, and bladder function scores. The highest possible score is 17 points. A lower score corresponds to greater dysfunction.
iii) Bazaz classification for dysphagia: According to the severity of swallowing difficulties, dysphagia was divided into 4 grades: none, mild, moderate, and severe.
iv) Other complications: Infection or injury of the surrounding tissues was evaluated.

### Follow-up

A minimum of 2 years of follow-up was required in this study. Considering the local medical insurance policy and affordability for the patients, imaging data were mainly obtained from cervical radiographs at the final follow-up. Computed tomography and MRI were only performed in patients requiring further clarification of the cause of injury.

### Statistical analysis

SPSS22.0 software was used for the statistical analysis. Continuous variables are expressed as the mean ± standard deviation. Independent sample *t* tests were performed to compare the relevant data between 2 different operative timing groups. The chi-square test was used to compare relevant data between three different fusion segment groups. *p* < 0.05 was considered statistically significant.

## Results

### Baseline patient characteristics and grouping

**Fig. 1:**
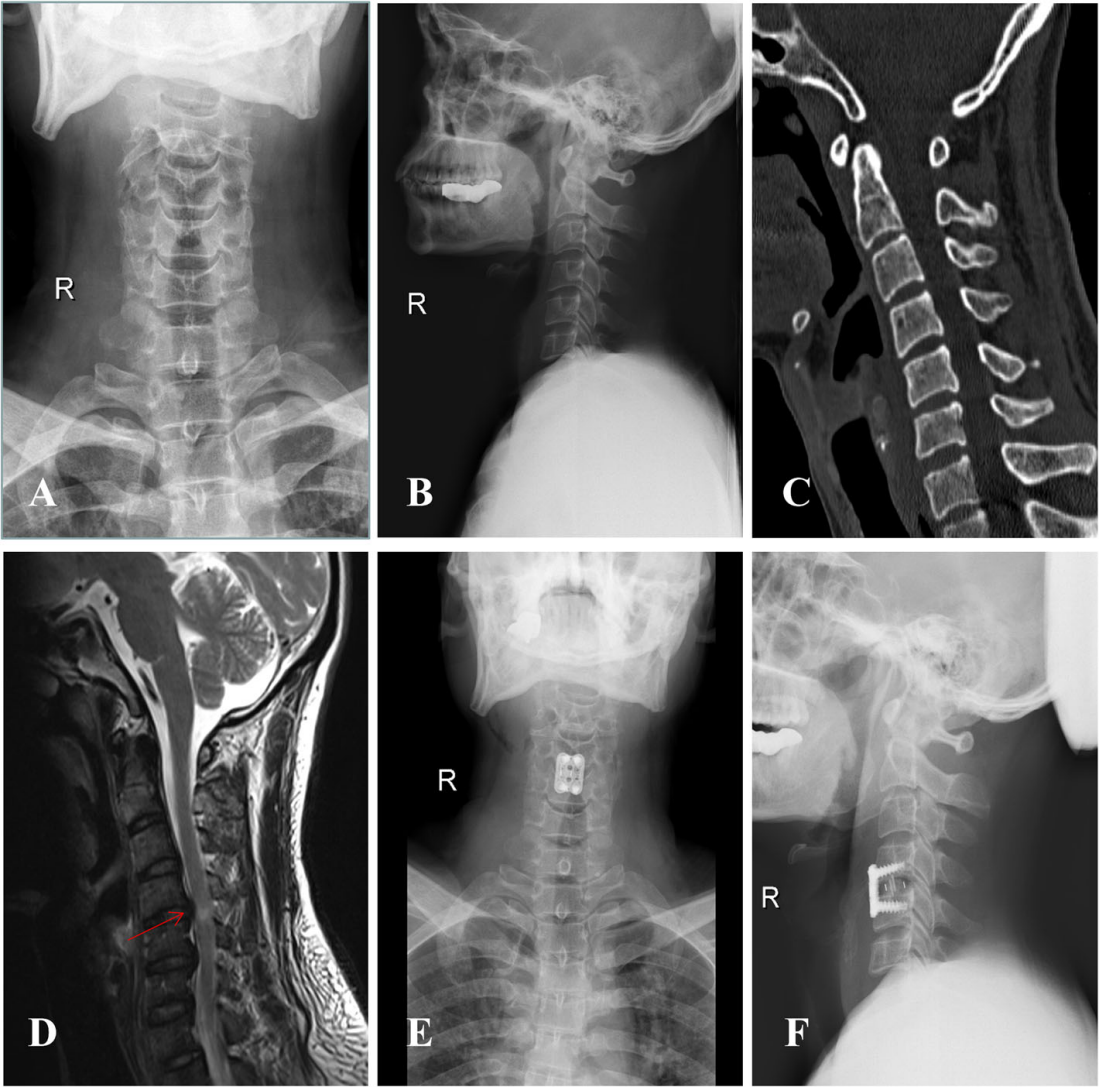
Imaging findings and operative radiographs for patient with cervical spinal cord injury without fracture and dislocation. **a** and **b** Preoperative cervical spine X-ray images with no obvious abnormal findings; **c** preoperative cervical computed tomography indicating no fracture or dislocation at the C4/5 segment; **d** preoperative cervical MRI, with the red mark showing cervical disc herniation, epidural spinal cord compression, and an abnormal spinal cord signal at the C4/5 segment; **e** and **f** postoperative cervical spine X-ray images showing cage and fixation devices in good positions after surgery

According to the above study protocol, a total of 78 patients (44 males and 34 females) were included in the study. No significant differences in age, gender, and cervical vertebral canal index were identified between the groups (*p* > 0.05). The general data of each group are shown in Table 1. Perioperative imaging data are shown in Fig. 1.

**Table 1:** The patients’ demographics of each group

Items	Lumbar segments	No.	Age (years)	Gender (M/F)	Cervical vertebral canal index
< 72 h	1 level	17	53.79 ± 11.49	10/7	0.91 ± 0.06
	2 levels	13	56.44 ± 13.01	6/7	0.93 ± 0.08
	3 levels	8	58.83 ± 12.95	5/3	0.84 ± 0.07
> 72 h	1 level	15	55.68 ± 11.75	9/6	0.92 ± 0.07
	2 levels	14	56.59 ± 12.09	8/6	0.91 ± 0.07
	3 levels	11	59.56 ± 11.86	6/5	0.82 ± 0.08

*No.* number, *M* male, *F* female, *h* hours

### Preoperative and postoperative ASIA and JOA scores for the early (within 72 h) and delayed (after 72 h) surgery groups

Thirty-eight patients underwent surgery within 72 h, and 40 patients underwent delayed surgery (after 72 h). The statistical results for the ASIA and JOA scores before and after surgery are shown in Table 2. Significant differences between the preoperative and postoperative ASIA and JOA scores were found in each group (*p* < 0.05). No significant differences in preoperative ASIA and JOA scores, and ASIA and JOA scores at 1 month after surgery were found between the two groups (*p* > 0.05). Over the course of the follow-up, the postoperative ASIA and JOA scores of both groups showed an upward trend. At 6 months (*p* < 0.05), 12 months (*p* < 0.05), and 24 months (*p* < 0.05) after surgery, the ASIA and JOA scores of the patients who received early ACDF were significantly better than those of the patients who underwent delayed surgery.

**Table 2:** Comparison of ASIA and JOA scores before and after surgery between early and delayed surgery groups

Items	Follow-up	< 72 h (*n* = 38)	> 72 h (*n* = 40)	*p*
ASIA score	Preoperative	49.81 ± 11.52^a^	51.33 ± 10.74^a^	> 0.05
	Postop. 1 month	62.13 ± 9.89	59.87 ± 12.03	> 0.05
	Postop. 6 months	78.56 ± 6.44	64.28 ± 7.39	< 0.05
	Postop. 12 months	81.77 ± 8.31	66.91 ± 9.27	< 0.05
	Postop. 24 months	85.87 ± 7.43	71.06 ± 10.91	< 0.05
JOA score	Preoperative	7.83 ± 1.72^a^	7.76 ± 1.72^a^	> 0.05
	Postop. 1 month	9.85 ± 1.73	9.78 ± 1.89	> 0.05
	Postop. 6 months	12.77 ± 1.64	10.35 ± 1.64	< 0.05
	Postop. 12 months	13.81 ± 1.46	10.90 ± 2.03	< 0.05
	Postop. 24 months	14.79 ± 1.35	12.27 ± 1.74	< 0.05

*ASIA* American Spinal Injury Association, *JOA* Japanese Orthopaedic Association, *Postop* postoperative, *h* hours

^a^Compared with the ASIA and JOA scores at postoperative 1, 6, 12, and 24 months after surgery in the same group, *p* < 0.05

### Preoperative and postoperative ASIA and JOA scores for patients requiring fusion of different numbers of segments

The 78 patients were divided into 3 groups according to the number of fused segments, with 32 patients in the single-segment fusion group, 27 patients in the double-segment fusion group, and 19 patients in the three-segment fusion group (Table 3). The postoperative ASIA and JOA scores of each segment group were significantly higher than the preoperative scores (*p* < 0.05). No significant differences were found in the preoperative ASIA and JOA scores, the postoperative ASIA, and JOA scores at 1 month, the postoperative JOA at 6 months among the three groups (*p* > 0.05). However, comparisons among the three groups revealed that the postoperative ASIA and JOA scores at 12 months and 24 months (*p* < 0.05) and ASIA score at 6 months (*p* < 0.05) were better in the single-segment and double-segment fusion groups than those in the three-segment fusion group.

**Table 3:** Comparison of ASIA and JOA scores before and after surgery between the groups of different fused segments

Items	Follow-up	1 level (*n* = 32)	2 levels (*n* = 27)	3 levels (*n* = 19)	*p*
ASIA score	Preoperative	51.08 ± 12.52 ^a^	48.97 ± 9.88^a^	50.16 ± 10.52^a^	> 0.05
	Postop. 1 month	63.13 ± 8.79	59.87 ± 12.03	62.73 ± 9.89	> 0.05
	Postop. 6 months	80.09 ± 6.86	78.91 ± 7.12	70.23 ± 6.72	< 0.05
	Postop. 12 months	81.21 ± 7.84	80.02 ± 8.93	69.52 ± 9.01	< 0.05
	Postop. 24 months	84.36 ± 7.58	82.91 ± 8.72	71.84 ± 8.20	< 0.05
JOA score	Preoperative	7.89 ± 1.86^a^	7.74 ± 1.59^a^	7.68 ± 1.68^a^	> 0.05
	Postop. 1 month	9.87 ± 2.01	9.79 ± 1.56	9.96 ± 1.86	> 0.05
	Postop. 6 months	11.47 ± 1.76	11.26 ± 1.61	11.04 ± 1.69	> 0.05
	Postop. 12 months	13.04 ± 2.31	12.94 ± 1.88	10.98 ± 1.81	< 0.05
	Postop. 24 months	13.87 ± 1.68	13.79 ± 2.09	11.85 ± 1.74	< 0.05

*ASIA* American Spinal Injury Association, *JOA* Japanese Orthopaedic Association, *Postop* postoperative

^a^Compared with the ASIA and JOA scores at postoperative 1, 6, 12, and 24 months after surgery in the same group, *p* < 0.05

### ASIA score, JOA score, and ASIA impairment scale under the interaction of the timing of surgery and the number of fused segments

Further comparison of the statistical differences in ASIA and JOA scores at 1, 6, 12, and 24 months (*p* < 0.05) after surgery showed that the patients who underwent early single-segment and double-segment fusion were more likely to achieve good functional recovery after surgery (Table 4). However, for the patients who underwent three-segment fusion, no significant differences were observed between the early and delayed surgery groups (*p* > 0.05).

**Table 4:** The results of ASIA and JOA scores under the interaction between the surgical timing and the number of the fused segments

Items	Follow-up	1 level		2 levels		3 levels	
		< 72 h (*n* = 17)	> 72 h (*n* = 15)	< 72 h (*n* = 13)	> 72 h (*n* = 14)	< 72 h (*n* = 8)	> 72 h (*n* = 11)
ASIA score	Postop. 1 month	64.14 ± 8.43	61.97 ± 7.91	60.38 ± 8.06	58.61 ± 7.83	63.97 ± 9.07	62.01 ± 8.75
	Postop. 6 months	84.56 ± 10.22	76.41 ± 11.37^a^	81.74 ± 9.84	73.85 ± 10.08^a^	69.85 ± 8.01	70.34 ± 7.82
	Postop. 12 months	86.22 ± 11.37	76.93 ± 10.24^a^	84.24 ± 9.37	74.63 ± 8.87^a^	70.72 ± 7.94	69.91 ± 8.27
	Postop. 24 months	87.37 ± 8.98	78.15 ± 10.77^a^	86.04 ± 9.11	75.06 ± 9.68^a^	72.14 ± 8.27	71.48 ± 9.06
JOA score	Postop. 1 month	9.95 ± 1.96	9.82 ± 2.07	9.75 ± 1.73	9.82 ± 1.47	9.83 ± 1.47	10.06 ± 2.14
	Postop. 6 months	12.11 ± 1.59	11.03 ± 1.75^a^	11.81 ± 1.60	10.36 ± 1.52^a^	11.17 ± 1.75	10.94 ± 1.69
	Postop. 12 months	13.83 ± 1.88	12.32 ± 2.01^a^	13.06 ± 1.94	11.90 ± 1.87^a^	12.41 ± 2.01	12.05 ± 1.77
	Postop. 24 months	14.25 ± 1.66	13.17 ± 1.42^a^	14.42 ± 1.932	12.78 ± 1.88^a^	12.91 ± 1.82	12.17 ± 1.90

*ASIA* American Spinal Injury Association, *JOA* Japanese Orthopaedic Association, *Postop* postoperative, *h* hours

^a^Compared with < 72 h group, *p* < 0.05

The ASIA impairment scale results (Table 5) showed that for the patients who underwent surgery, postoperative neurological function was significantly better than preoperative neurological function (*p* < 0.05). For patients undergoing single- or double-segment ACDF, early surgery (within 72 h) achieved better postoperative neurological function recovery than delayed surgery. However, for patients undergoing three-segment fusion, there was no significant difference in the ASIA impairment scale grade between early and delayed surgery (*p* > 0.05).

**Table 5:** The results of ASIA impairment scale under the interaction between the surgical timing and the number of the fused segments

Items	Follow-up	Preoperative	Postop. 1 m	Postop. 6 m	Postop. 12 m	Postop. 24 m
1 level	< 72 h (*n* = 17)	A, 1; B, 3; C, 5; D, 8	A, 1; B, 2; C, 5; D, 9	A, 0; B, 2; C, 6; D, 9	A, 0; B, 1; C, 5; D, 11	A, 0; B, 1; C, 3; D, 13
	> 72 h (*n* = 15)	A, 2; B, 3; C, 4; D, 6	A, 2; B, 3; C, 3; D, 7	A, 2; B, 3; C, 2; D, 8	A, 1; B, 3; C, 3; D, 8	A, 1; B, 3; C, 3; D, 8
2 levels	< 72 h (*n* = 13)	A, 2; B, 3; C, 3; D, 5	A, 2; B, 3; C, 2; D, 6	A, 2; B, 2; C, 2; D, 7	A, 1; B, 1; C, 3; D, 8	A, 0; B, 2; C, 2; D, 9
	> 72 h (*n* = 14)	A, 2; B, 2; C, 3; D, 7	A, 2; B, 2; C, 3; D, 7	A, 2; B, 2; C, 2; D, 8	A, 2; B, 2; C, 2; D, 8	A, 1; B, 2; C, 3; D, 8
3 levels	< 72 h (*n* = 8)	A, 2; B, 3; C, 2; D, 1	A, 2; B, 3; C, 2; D, 1	A, 2; B, 2; C, 2; D, 2	A, 2; B, 2; C, 2; D, 2	A, 1; B, 2; C, 3; D, 2
	> 72 h (*n* = 11)	A, 2; B, 4; C, 2; D, 3	A, 2; B, 4; C, 1; D, 4	A, 2; B, 4; C, 1; D, 4	A, 2; B, 3; C, 1; D, 5	A, 2; B, 1; C, 2; D, 6

*ASIA* American Spinal Injury Association, *Postop* Postoperative, *m* month(s), *h* hours

### Complications

Seven patients had surgery-related complications, including 2 patients with infection (1 patient with pneumonia and 1 patient with urinary tract infections) and 1 patient with postoperative hoarseness. All these patients were cured and discharged from the hospital after appropriate interventions. Four cases of dysphagia were identified in the three-segment group at the 6-month follow-up. The incidence was significantly higher in the three-segment group than in the other two groups.

## Discussion

### Main findings

To the best of our knowledge, this is the first clinical study to investigate the effect of the interaction between surgical timing and the number of fused segments on the prognosis of patients with CSCIWFD who underwent ACDF. This study showed that ACDF with different numbers of fused segments performed at different times can lead to varying degrees of functional improvement in CSCIWFD patients, and early ACDF (within 72 h) is associated with a better prognosis compared with delayed ACDF (after 72 h). Comprehensive analysis of the interaction between the timing of surgery and the number of fused segments indicated that early surgery can achieve superior postoperative functional recovery in patients undergoing single- or double-segment fusion. For patients undergoing three-segment fusion, both early and delayed surgery provided effective neurological recovery, but early surgery did not show an advantage over delayed surgery.

### The effect of surgical timing on patients with CSCIWFD

Persistent mechanical compression caused by disc fragments and hematoma can substantially reduce blood perfusion and lead to neuronal death around the lesion due to ischemia [11]. For patients with SCI, mechanical compression combined with progressive destruction of nerve tissue caused by secondary cell damage [12] is often the main cause of a poor prognosis in such patients. Several preclinical studies [13, 14] have shown that decompression surgery can improve functional recovery in animal models with SCI-induced compression. Prevention of secondary injury in animal models is also closely related to the timing of surgical decompression [15].

Considering the negative effects of mechanical compression on SCI, several clinical studies investigating the efficacy of decompression surgery for SCI have been published. However, the results of these studies are controversial. Early observational studies led by Wagner and Chehrazi [16] showed no difference in postoperative neurological recovery among patients with cervical SCI who underwent decompression surgery within 8 h versus 9–48 h. In addition, Marshal et al. [17] abandoned early decompression surgery and advocated delayed surgery for patients with cervical SCI due to neurological deterioration observed within 5 days after early surgery. Early-stage studies have tended to conclude that early surgery is not beneficial for patients with cervical SCI or that the benefit is not significant compared with delayed surgery. However, with the development of spinal surgery, recent preclinical studies based on animal models have obtained theoretical evidence supporting early decompression surgery for patients with cervical SCI. To more scientifically and accurately evaluate the impact of early surgery on the prognosis of patients with cervical SCI, Fehlings et al. [18] performed a multicenter randomized trial. Their study showed that emergency decompression surgery within 24 h after cervical SCI did improve neurological recovery. Similar results have been reported in subsequent studies [6, 19]. Unfortunately, due to considerable differences in surgical timing in different studies, although consistent results have been obtained, uniform and clear evidence supporting a specific early surgical time limit is still lacking. Therefore, defining an appropriate time window for early surgery remains challenging. Level 2 evidence shows that early surgery within 24 h is safe and effective [18]. However, completing the necessary medical examinations, organizing a well-equipped surgical team, and developing timely and effective backup plans within 24 h are often difficult tasks. Under the current medical system in southern China, most patients cannot undergo early surgery in qualified hospitals within 24 h. Therefore, based on careful consideration, the time limit for early surgery in this study was 72 h. This study suggests that ACDF is a safe and effective treatment for CSCIWFD. Moreover, patients who underwent surgery within 72 h had a significantly better prognosis than those who underwent delayed surgery. Our results reinforce the conclusion that early surgery has a positive effect on neurological recovery in patients with cervical SCI and suggests that the time limit for early surgery can be defined as 72 h, especially for patients who cannot undergo earlier surgery.

### The effect of the number of fused segments on patients with CSCIWFD

Previous studies have focused on the impact of the number of fused segments on ASD [20]. In this study, patients who had different numbers of fused segments achieved different degrees of functional recovery after surgery. However, during the 1-year follow-up and beyond, short-segment fusion (within 2 segments) was associated with a better prognosis in both the early surgery and delayed surgery groups. The prognosis of CSCIWFD patients is closely related to the degree of cervical SCI. Although the preoperative ASIA and JOA scores were not significantly different between the short- and long-segment groups, the patients who underwent three-segment ACDF often exhibited a greater degree of disc degeneration. The injuries in the three-segment group were mainly high-energy injuries caused by high-impact forces. Nerve tissue repair is a slower process. Therefore, extensive multiple-segment injuries require more recovery time and often result in a limited extent of recovery, which may explain why the postoperative ASIA and JOA scores at 6 and final follow-up in the three-segment fusion group were worse than those in the short-segment groups. Considering the timing of surgery, our findings tend to advocate early ACDF in patients with single- and double-segment injuries under adequate preoperative preparation. For patients with three-segment injuries, surgery is still required, but the timing of surgery should be determined according to the patient’s individual situation.

### Complications

Complications are often an important aspect when evaluating surgical outcomes. As a well-accepted and effective surgical approach, ACDF is widely used in clinical practice [21]. Postoperative dysphagia is a relatively common complication of ACDF in the short term. The incidence of dysphagia after ACDF is not consistent across studies, ranging from 3 to 21% [22]. In this study, we found that the incidence of dysphagia at 6 months after surgery was 5.13% (4/78), which is lower than the incidence of dysphagia (13–21%) reported by Riley et al. [23] at 1 year after surgery. In contrast, the incidence of postoperative dysphagia was all from in the patients who underwent three-segment fusion. Our results reinforce the conclusions of other studies that multisegment surgery is an important factor for postoperative dysphagia [21, 24]. Reducing the traction time of the neck muscles and the esophagus during surgery and using a static retractor or a small, thin bone plate are effective measures to reduce postoperative dysphagia. The surgeon should carefully monitor surgery-related dysphagia in the perioperative period.

### Limitations

Similar to other clinical studies, this study has some limitations. First, the limited number of patients in each group may reduce the statistical power of the results to some extent. Moreover, due to limitations of the local medical system, many patients could not receive early surgery because of multiple referrals. Therefore, this study could not stratify the data regarding surgical timing. However, strong evidence indicates that surgery within 72 h can be defined as early surgery in this study. Another limitation is that some patients received different treatments in other hospitals before surgery, and the injury mechanism of the patients and the severities of cervical SCI were not identical. In addition, although hemodynamic monitoring is a very important factor in the prognosis of these patients, whether operated or not operated, we were not authorized to obtain the actual data for the patients because the physicians’ orders and the electronic medical records were sealed 3 months after the patients were discharged from the hospital. The combined effects of these factors may have an impact on the results of this study.

## Conclusions

In summary, ACDF is a safe and effective treatment for CSCIWFD. Both early and delayed ACDF can benefit these patients. For patients with single- or double-segment injuries, early (within 72 h) ACDF is associated with a more satisfactory prognosis. Although the efficacy of three-segment ACDF is not superior to that of short-segment fusion, ACDF still has a positive effect on the prognosis of CSCIWFD patients, and the timing of surgery should be based on each patient’s individual situation. Due to the limitation of the small sample size, we cautiously recommend that 72 h can be defined as an appropriate time limit for early surgery for these patients in regions where earlier surgery cannot be provided by the current diagnosis and treatment system. Large-sample-sized, multicenter, high-quality studies are expected to obtain a higher level of evidence.

## Data Availability

The datasets generated and analyzed during the current study are available from the corresponding author on reasonable request.

## Abbreviations

ACDF: Anterior cervical decompression and fusion
ASD: Adjacent segment degeneration
ASIA: American Spinal Injury Association
CSCIWFD: Cervical spinal cord injury without fracture and dislocation
JOA: Japanese Orthopaedic Association
MRI: Magnetic resonance imaging
SCI: Spinal cord injury
